# The Added Role of Diffusion-Weighted Magnetic Resonance Imaging in Staging Uterine Cervical Cancer

**DOI:** 10.7759/cureus.75707

**Published:** 2024-12-14

**Authors:** Ramona A Rizescu, Iulia A Salcianu, Alexandre Ionescu, Alexandru Serbanoiu, Radu T Ion, Lucian M Florescu, Gheorghe Iana, Ana M Bratu, Ioana A Gheonea

**Affiliations:** 1 Doctoral School of Medicine, University of Medicine and Pharmacy of Craiova, Craiova, ROU; 2 Department of Radiology, Coltea Clinical Hospital, Bucharest, ROU; 3 Department of Radiology, University of Medicine and Pharmacy "Carol Davila", Bucharest, ROU; 4 Department of Radiology, The University Emergency Hospital Bucharest, Bucharest, ROU; 5 Department of Anatomy, University of Medicine and Pharmacy "Carol Davila", Bucharest, ROU; 6 Department of Radiology, University of Medicine and Pharmacy of Craiova, Craiova, ROU

**Keywords:** apparent diffusion coefficient (adc), diffusion-weighted imaging (dwi), international federation of gynaecology and obstetrics (figo) staging, pathological staging, uterine cervical cancer

## Abstract

Background: Cervical cancer is considered one of the most common gynecological malignancies with an increased incidence in developing countries. Magnetic resonance imaging (MRI) plays a valuable role in staging cervical cancer and providing valuable information necessary for selecting the appropriate treatment plan, while closely correlating with the prognosis of the patient.

Objective: The aim of this study is to assess the diagnostic value of diffusion-weighted imaging (DWI) in the preoperative loco-regional staging of cervical carcinoma. Our purpose is to establish apparent diffusion coefficient (ADC) values of cervical carcinoma compared with normal cervical tissue and their variability based on different pathological characteristics of the lesions.

Material and methods: It is a retrospective analysis of 57 patients diagnosed with cervical cancer, who underwent MRI examinations. The study evaluated the aspect of the lesions on T2-weighted imaging, DWI, ADC maps, and pre- and post-contrast T1-weighted imaging with fat saturation.

Results: The ADC mean values ranged between 0.63 × 10^−3^ mm^2^/second and 0.99 × 10^−3^ mm^2^/second (mean 0.79) for tumoral tissue and 1.33 × 10^−3^ mm^2^/second and 1.74 × 10^−3^ mm^2^/second (mean 1.59) for surrounding non-affected cervical tissue. The ADC mapping showed a decreasing trend with the increased sizes of the tumors (p<0.001). The ADC mean showed lower values with increased International Federation of Gynecology and Obstetrics (FIGO) stage of the tumors. The ADC mean value for cases that had spread to other organs (IVA+IVB) was significantly lower than that of the early stages (IB1 + IB2 + IIA2), stage IIB, and stages IIIA+IIIC1+IIIC2 (p<0.001). The ADC mean value of stage III disease was significantly lower than that of stage IIB, respectively early stages (p<0.001). The ADC mean value of the stage IIB tumor was significantly lower than that of the early stages (p<0.001). The differences in ADC mean values based on the histopathological type and differentiation grade were not statistically significant. The ADC mean value of the cases with positive pelvic lymph nodes was significantly lower than in those with negative lymph nodes (p<0.001).

Conclusion: ADC mean values of cervical carcinoma are significantly lower than those from unaffected uterine tissue and they also correlate with the severity of the disease. The advancements and additional capabilities DWI can bring are the elements of interest in this article. Using DWI means a more accurate capability in diagnosing cervical cancer, providing a compelling argument for its integration into standard clinical practice. This study discusses the quantitative imaging parameters of DWI such as ADC values, which can provide objective measurements for tumor evaluation. These parameters can be standardized and used across different institutions, enhancing the reproducibility and reliability of imaging findings.

## Introduction

Uterine cervical cancer is the fourth most common malignancy in female patients with an incidence of 660,000 women/year and causing 350,000 deaths in 2022, according to the World Health Organization (WHO) [1]. There is a significant increase in the incidence in developing countries. Although it is one of the most preventable and treatable malignant diseases, 40% of the patients present with locally advanced disease, chemotherapy being the standard curative care [2,3]. The International Federation of Gynecology and Obstetrics (FIGO) staging system updated in 2021 has an added value on the prognosis and treatment of cervical cancer [4].

The role of magnetic resonance imaging (MRI) in gynecological malignancies has evolved, as it is the gold standard used for diagnosing and staging primary cervical cancer. It accurately evaluates the extension of cervical cancer as it can provide an exact measurement of the tumor size and parametrial invasion [5]. As it has a superior soft tissue delineation and due to the multiplanar image acquisition, MRI is an integral part of local staging cervical cancer. It has a major role in the selection of the correct surgical approach, whether conization with uterus-preserving surgery is applicable or total hysterectomy is recommended. In addition, MRI has an important role in monitoring patient response and detecting recurrence, as well as complications that may appear. It has proved to be a cost-effective method by limiting further diagnostic tests or surgical procedures [6,7].

Diffusion-weighted imaging (DWI) is widely used in diagnosing acute stroke [8]. The latest development in MRI technology has led to a reduction in the artifacts that used to affect the image interpretation and DWI has been extensively used in imaging of the abdomen and pelvis [9-11]. DWI is used for calculating apparent diffusion coefficient (ADC) maps and the quantification of ADC values is used in differentiating malignant from benign lesions [12].

T2 weighted images have a superior soft tissue contrast and are widely used for evaluating tumor extension and staging cervical cancer. For extensive tissue characterization, such as differentiating post-therapeutic changes and residual tumors, DWI is an important tool. DWI shows restriction in water mobility in tissue. Malignant tumoral tissue changes the water mobility due to the microenvironmental pathologic conditions and can be an early indicator of chemotherapy treatment response. ADC mappings can assess microenvironmental changes in target areas and can be used to differentiate between complete, partial, and non-respondent treatment outcomes. The response can be predicted by the changes in ADC values early during treatment (chemoradiotherapy or chemotherapy). An increase in ADC values is often correlated to a positive response due to treatment-induced cell death and reduced cellularity [13]. The accurate preoperative assessment of lymph nodes has an important role in choosing the appropriate treatment plan, in case adjuvant radiation therapy is recommended. Studies have shown that malignant lymph nodes presented decreased ADC values when compared to benign lymphadenopathies [14].

The purpose of this study is to estimate the additional role of DWI in the assessment of cervical carcinoma and to emphasize the role of the different acquisition planes in order to accurately stage the lesions.

## Materials and methods

This was a retrospective study of the database of Coltea Clinical Hospital, Bucharest, Romania, from January 2019 to May 2024, which included 57 female patients, aged 33-82 years, who presented with abnormal vaginal bleeding, were diagnosed with cervical cancer, and underwent MRI examinations. Thirty patients (52.6%) were in menopausal state at the time of diagnosis. As this was a retrospective study, ethical review and approval were waived.

The clinical data, imaging reports, and histopathological results were collected from the electronic medical records of Coltea Clinical Hospital. Patient data are gathered and kept in an anonymized and centralized Microsoft Excel spreadsheet (Microsoft Corporation, Redmond, Washington, United States).

Imaging protocol

Patients were positioned in a supine position with phased-array abdominal coils and the MRI acquisitions were carried out using 1.5 T scanners, with free breathing. In all cases, the creatinine level was checked in order to prevent renal injury caused by contrast media administration. The machine used was Magnetom Sola 1.5T (Siemens Healthineers AG, Erlangen, Germany).

The MRI protocol included: T2-weighted fast spin-echo imaging in sagittal plane (TR 5060 ms, TE 100 ms, slice thickness of 3 mm, distance factor 10%, field of vision of 250 mm, matrix 384x384), T2-weighted fast spin-echo imaging aligned coronally to the longitudinal axis of the uterine body (TR 3840 ms, TE 100 ms, slice thickness of 3 mm, distance factor 10%, field of vision of 200 mm, matrix 640*640), T2-weighted fast spin-echo imaging aligned axially to the longitudinal axis of the uterine body (TR 4500 ms, TE 100 ms, slice thickness of 3 mm, distance factor 10%, field of vision of 200 mm, matrix 640x640), T1-weighted fast spin-echo imaging in axial plane (TR 600 ms, TE 20 ms, slice thickness of 4 mm, distance factor 30%, field of vision of 320 mm, matrix 640x640), DWI obtained in the axial plane by using a single shot echo-planar imaging sequence with b values of 50, 400, and 800 s/mm^2^ (the ADC map is automatically calculated based on DWI), and T1-weighted images with fat suppression (TR 5 ms, TE 2.5 ms, slice thickness of 3 mm, distance factor 20%, field of vision of 380 mm, matrix 320x260), before and after administration of 10 mL of gadolinium and 20 mL saline. 

Image analysis

The MRI aspect of the lesions was evaluated based on their size, signal intensity, and contrast enhancement. The extension to other pelvic organs was assessed, as well as the presence of infiltrated pelvic lymph nodes. The staging of cervical carcinoma was performed using the T2-weighted, DWI, and post-contrast images, and it was based on the FIGO staging analysis.

The interpretation of DWI images included both a qualitative and a quantitative analysis. Malignant lesions displayed restricted diffusion with high signal intensity on DWI and low signal intensity on ADC maps, while areas that showed reduced signal intensity could indicate necrosis. Regarding the quantitative analysis of DWI, the tumors were manually identified and then the mean ADC values (× 10-3 mm) were obtained on the workstation.

The MR images were analyzed and the measurements were made by two radiologists with more than five years’ experience in body MRI. The maximum diameters of the tumors were measured in each of the three planes two times and the average maximum value was obtained. A region of interest (ROI) was manually placed on the ADC map along the peripheral aspect of the tumor area in three different places in order to obtain a mean ADC value. It was placed on the tumoral tissue in order to avoid areas of heterogenous signal such as necrosis, cystic degeneration, and bleeding. An ROI was also placed on the surrounding normal cervical tissue. 

Data analysis

Data analysis and graphical representations were conducted using IBM SPSS Statistics for Windows, Version 26.0 (Released 2019; IBM Corp., Armonk, New York, United States) and Microsoft Excel (version 16.85). An independent paired samples t-test was used to compare and analyze the difference between the ADC values of the surrounding normal tissue and the ADC values of the tumoral tissue. When more than two groups were subjected to analysis, an analysis of variance (ANOVA) was the statistical test of choice. An independent samples t-test was performed when comparing the tumoral ADC values in case of lymph node metastasis. Results were considered significant if the P value was ≤ 0.05.

## Results

A total of 57 women with cervical carcinoma confirmed by histopathological analysis, aged 33-82 years (mean 56 years) were included. Postmenopausal vaginal bleeding was the main symptom the patients presented with (38 cases, 52.6%). Women in the reproductive age group presented with irregular vaginal bleeding (19 patients, 33.4%), while perimenopausal women presented with irregular and heavy vaginal bleeding (eight patients, 14%).

**Figure 1:**
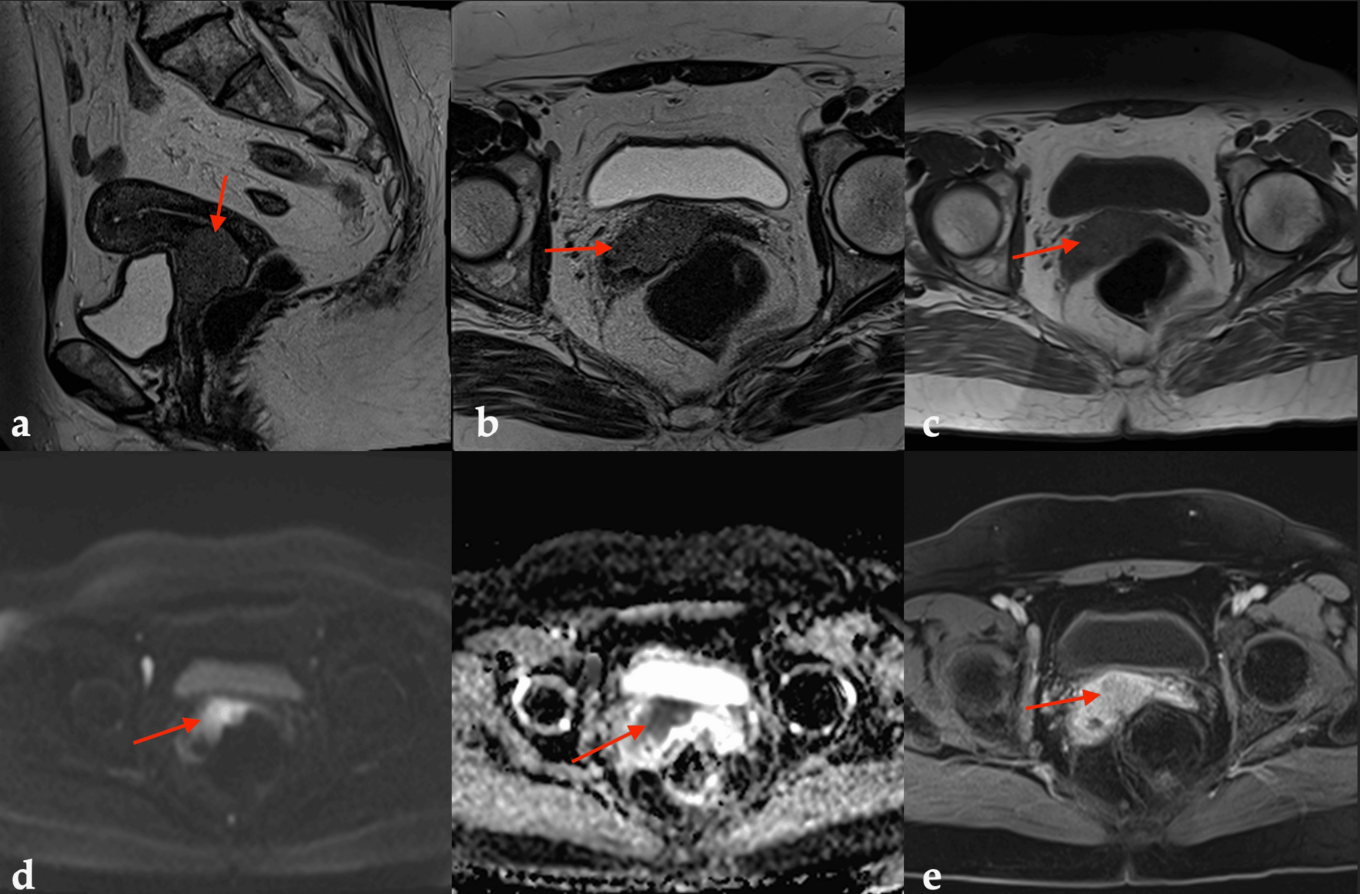
Sixty-four-year-old female patient complaining of post-menopausal bleeding. Pathology revealed invasive non-keratinizing squamous cell carcinoma. (a, b) Sagittal and axial T2WI showed a hyperintense lesion; (c) Axial T1WI revealed an isointense cervical lesion; (d) High signal on DWI and low ADC levels. ADC mean value was (0.63 × 10−3 mm^2^/second); (e) T1 FS postcontrast axial image with heterogeneous enhancement. Red arrows are pointing to the lesion. T2WI: T2 weighted imaging; DWI: diffusion-weighted imaging; ADC: apparent diffusion coefficient; T1 FS: T1 weighted fat-saturated

**Figure 2:**
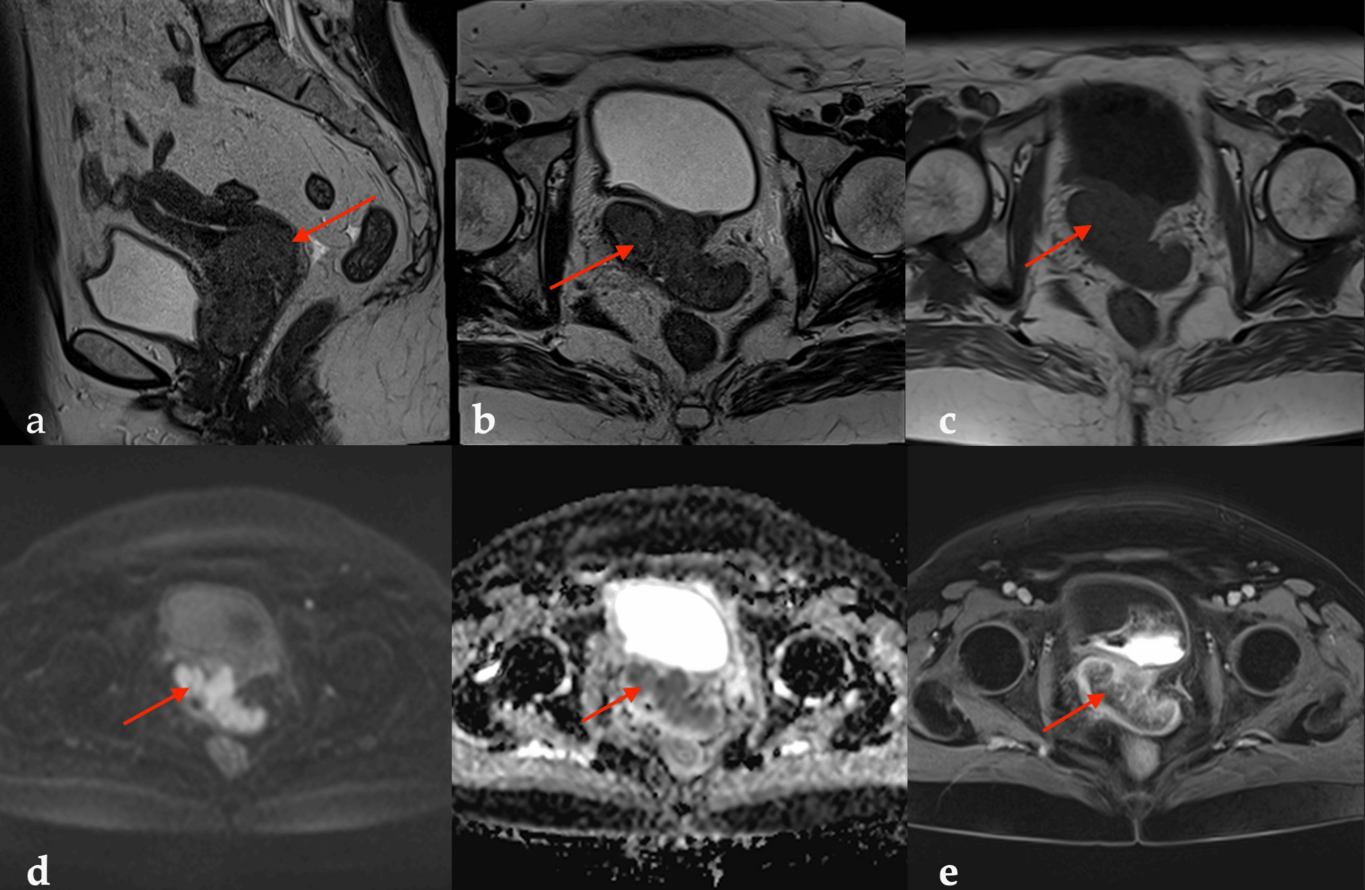
Sixty-four-year-old female patient complaining of post-menopausal bleeding. Pathology revealed invasive keratinizing squamous cell carcinoma. (a, b) Sagittal and axial T2WI showed a heterogenous lesion; (c) Axial T1WI revealed an isointense cervical lesion; (d) High signal on DWI and low ADC levels. ADC mean value was (0.65 × 10−3 mm^2^/second); (e) T1 FS postcontrast axial image with heterogeneous enhancement. Red arrows point to the lesion. T2WI: T2 weighted imaging; DWI: diffusion-weighted imaging; ADC: apparent diffusion coefficient; T1 FS: T1 weighted fat-saturated

**Figure 3:**
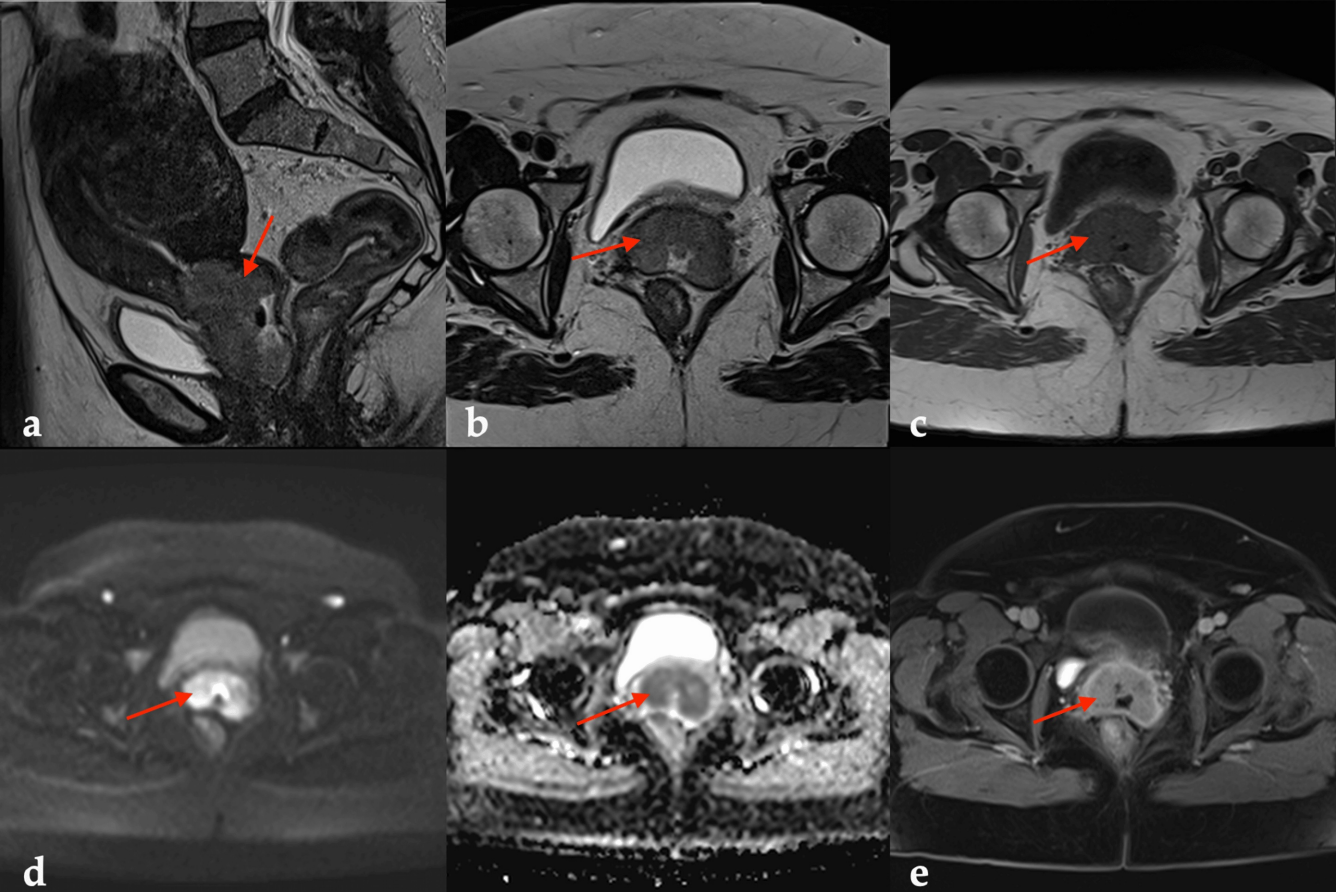
Fifty-three-year-old female patient complaining of post-menopausal bleeding. Pathology revealed endocervical adenocarcinoma. (a, b) Sagittal and axial T2WI showed hyper intense lesion; (c) Axial T1WI revealed isointense cervical lesion; (d) High signal on DWI and low ADC levels. ADC mean value was (0.78 × 10−3 mm^2^/second); (e) T1 FS postcontrast axial image with heterogeneous enhancement.  Red arrows point to the lesion. T2WI: T2 weighted imaging; DWI: diffusion-weighted imaging; ADC: apparent diffusion coefficient; T1 FS: T1 weighted fat-saturated

The pathology of the evaluated lesions was squamous cell carcinoma (SCC) in 50 patients and endocervical adenocarcinoma in seven patients. In the 50 SCC cases, three were noted as in situ carcinomas (one with CIN1 and two with CIN3), 34 were invasive keratinizing (seven well-differentiated cases: grade G1; 10 moderately differentiated cases: grade G2; 17 poorly differentiated cases: grade G3), and 13 were invasive non-keratinizing (one was grade G1; seven were grade G2; five were grade G3). In seven endocervical adenocarcinomas, one was in situ, one was grade G1, two were grade G2, and three were grade G3 (Figures 1-3).

The FIGO staging of the included patients showed that 21% of patients were stage IB1 and IB2, 5.3% were stage IIA2, and 12.3% were stage IIB. Stages IIIA and IIIC were demonstrated in 1.8% and 21.1% of patients, respectively, and 22.8% of the patients were stage IVA and 15.8% of the patients were IVB (Table 1).

**Table 1:** FIGO staging of the included patients (N=57) FIGO: International Federation of Gynecology and Obstetrics

FIGO Stage	Number of patients	Percentage
IB1	6	10.5%
IB2	6	10.5%
IIA2	3	5.3%
IIB	7	12.3%
IIIA	1	1.8%
IIIC1	11	19.3%
IIIC2	1	1.8%
IVA	13	22.8%
IVB	9	15.8%

We evaluated whether the tumor has spread to the surrounding tissues and organs. Parametrial infiltration was noted in 37 cases (64.9%). The tumor presented an extension to the vagina in 32 cases (56.1%). Extension to the uterine corpus was noted in 29 cases (50.8%). The tumor infiltrated the posterior wall of the bladder in 17 cases (29.8%) and the rectum in nine cases (15.8%). MRI aspect of likely metastatic lymph nodes was noted in 30 cases (52.6%).

The aspect of the cervical tumors on T2-weighted images showed a lower signal than the adjacent tissue in 17 patients (29.8%), intermediate signal in 18 patients (31.6%), higher signal in six patients (10.5%), and heterogenous signal in 16 patients (28.1%). The tumor contrast enhancement was low in 29 cases (50.9%) and heterogenous in 28 cases (49.1%). The maximum diameter of the tumor was noted and in six cases (10.5%), it was less than 20 mm, 21-40 mm in 15 cases (26.3%), 41-60 mm in 21 cases (36.8%), and over 60 mm in 15 cases (26.3%). 

The ADC values for the tumoral tissue and normal surrounding uterine tissue were measured. Values varied between 0.63 × 10−3 mm^2^/s and 0.99 × 10−3 mm^2^/second (mean 0.79) for tumoral tissue and 1.33 × 10−3 mm^2^/second and 1.74 × 10−3 mm^2^/second (mean 1.59) for non-affected cervical tissue (p<0.001) (Tables 2, 3).

**Table 2:** Mean ADC value of tumoral tissue and normal cervical tissue. ADC: apparent diffusion coefficient

	Mean	N
Tumoral tissue ADC value	0.7933	57
Normal tissue ADC value	1.5935	57

**Table 3:** Comparison of ADC mean value between tumoral tissue and normal cervical tissue. ADC: apparent diffusion coefficient

	Mean	Standard Deviation	p
Tumoral tissue ADC value versus normal tissue ADC value	-0.80018	0.10746	<0.001

The ADC mapping showed a decreasing trend with the increased size of the tumors. The ADC mean value for tumors larger than 60 mm was significantly lower than for those that measured 41-60 mm, 21-40 mm, and less than 20 mm (p<0.001) (Tables 4, 5).

**Table 4:** Mean ADC mean value based on the size of the tumor. ADC: apparent diffusion coefficient

Size	N	Mean	Std. Deviation	Min	Max
<21 mm	6	0.9217	0.06338	0.81	0.98
21-40 mm	15	0.8447	0.09257	0.64	0.99
41-60 mm	21	0.7571	0.07370	0.63	0.90
>60 mm	15	0.7413	0.08228	0.63	0.88
Total	57	0.7933	0.09909	0.63	0.99

**Table 5:** Comparison of ADC mean value based on the size of the tumor. ADC: apparent diffusion coefficient

	Sum of squares	df	Mean Square	p
Between groups	0.206	3	0.069	<0.001
Within groups	0.343	53	0.006	
Total	0.550	56		

The ADC mean showed lower values with increased FIGO stage of the tumor. The ADC mean value for cases that had spread to other organs (IVA+IVB) was significantly lower than that of the early stages (IB1 + IB2 + IIA2), stage IIB, and stages IIIA+IIIC1+IIIC2 (p<0.001). The ADC mean value of stage III disease was significantly lower than that of stage IIB, early stages (p<0.001). The ADC mean value of stage IIB tumor was statistically significantly lower than that of early stages (p<0.001) (Tables 6, 7).

**Table 6:** Mean ADC value based on the FIGO stage of the tumor. ADC: apparent diffusion coefficient; FIGO: International Federation of Gynecology and Obstetrics

FIGO stage	N	Mean	Std. Deviation	Min	Max
IB1+IB2+IIA2	15	0.8747	0.07160	0.74	0.98
IIB	7	0.8529	0.09639	0.73	0.99
IIIA+IIIC1+IIIC2	13	0.7569	0.08128	0.63	0.90
IVA+IVB	22	0.7405	0.08174	0.63	0.91
Total	57	0.7933	0.09909	0.63	0.99

**Table 7:** Comparison of ADC mean value based on the FIGO stage of the tumor. ADC: apparent diffusion coefficient; FIGO: International Federation of Gynecology and Obstetrics

	Sum of Squares	df	Mean Square	p
Between Groups	0.203	3	0.068	<0.001
Within Groups	0.347	53	0.007	
Total	0.550	56		

The differences in ADC mean values based on the histopathological type and differentiation grade were not statistically significant (Tables 8-11).

**Table 8:** Mean ADC value based on the histopathological type of the tumor. SCC: squamous cell carcinoma; ADC: apparent diffusion coefficient

Histopathological type	N	Mean	Std. Deviation	Min	Max
SCC in situ	3	0.7867	0.09074	0.72	0.89
Endocervical adenocarcinoma	7	0.8614	0.08533	0.78	0.98
Invasive keratinizing SCC	34	0.7815	0.09708	0.63	0.99
Invasive non keratinizing SCC	13	0.7892	0.10843	0.63	0.95
Total	57	0.7933	0.09909	0.63	0.99

**Table 9:** Comparison of ADC mean value based on the histopathological type of the tumor. SCC: squamous cell carcinoma; ADC: apparent diffusion coefficient

	Sum of Squares	df	Mean Square	p
Between Groups	0.038	3	0.013	0.285
Within Groups	0.512	53	0.010	
Total	0.550	56		

**Table 10:** Mean ADC value based on the histopathological grade of the tumor. ADC: apparent diffusion coefficient

Histopathological grade	N	Mean	Std. Deviation	Min	Max
CIN1+CIN3	4	0.8325	0.11786	0.72	0.97
G1	9	0.8322	0.09203	0.65	0.95
G2	19	0.8089	0.09712	0.63	0.95
G3	25	0.7612	0.09584	0.63	0.99
Total	57	0.7933	0.09909	0.63	0.99

**Table 11:** Comparison of ADC mean value based on the histopathological grade of the tumor. ADC: apparent diffusion coefficient

	Sum of Squares	df	Mean Square	p
Between Groups	0.050	3	0.017	0.163
Within Groups	0.500	53	0.009	
Total	0.550	56		

The ADC mean value of the cases with likely metastatic pelvic lymph nodes was significantly lower than that of the cases with negative lymph nodes (p<0.001) (Tables 12, 13).

**Table 12:** Mean ADC value based on the presence of pelvic lymph node metastasis. ADC: apparent diffusion coefficient

Lymph nodes metastasis		N	Mean	Std. Deviation
ADC mean value	negative	27	0.8530	0.08575
	positive	30	0.7397	0.07797

**Table 13:** Comparison of ADC mean value based on the presence of pelvic lymph node metastasis. ADC: apparent diffusion coefficient

		Levene's Test for Equality of Variances		t-test
		F	p	p
ADC mean value	Equal variances assumed	0.084	0.774	<0.001
	Equal variances not assumed			<0.001

## Discussion

Cervical cancer is a solid tumor that consists of atypical cells with high nuclear-cytoplasmic ratios that are densely distributed, reducing the free movement of water molecules at the microscopic level [15]. As a result, DWI has proved to be an important tool in detecting and staging cervical cancer. It has been showed that cervical cancer tissue shows restricted diffusion and low ADC values when compared to normal cervical tissue. It has been demonstrated in other studies that DWI offers a precise delineation of the tumor margins and reduces the rate of overestimating the volume of the lesion [16-18]. In T2-weighted images, the tumor is mostly described as a well-delineated mass with intermediate signal intensity compared to the T2 hypointense cervical stroma. However, there are cases where there is diffusely infiltrating carcinoma and the conventional MRI protocol has a lower accuracy in delineating the tumor, therefore impacting the correct staging [19]. DWI is also evaluated in an attempt to predict response to treatment. Tumors with higher ADC when diagnosed are usually more necrotic and cystic and, therefore, more hypoxic. In account of these factors, these tumors may be less responsive to chemotherapy [18]. Chemotherapy and radiation therapy induce cellular necrosis and apoptosis that lead to lysis and increased water diffusion. As a result, the response to treatment can be assessed early post-radiation/chemotherapy by DWI even if the tumor aspect suffers no visible change [20].

Even though there have been significant improvements in screening and prevention of cervical carcinoma, it continues to be a major cause of morbidity and mortality worldwide. The use of advanced imaging methods, especially MRI, has been encouraged in order to increase the accuracy of staging, choose the optimal treatment plan, and detect recurrence [21]. There is a wide variety of literature that emphasizes the added value of MRI in the initial evaluation of patients diagnosed with cervical cancer. MRI provides extensive details of the lesions due to the multiplanar and multiparametric acquisitions, thus staging and aiding the management of the case, outweighing the costs [22,23].

MRI has an important role in distinguishing early-stage disease that can be managed surgically from locally advanced or late-stage disease that requires chemoradiotherapy. MRI also assesses prognostic factors such as the size of the tumor, parametrial infiltration, pelvic wall invasion, nearby organ invasion, and lymph node metastases [24,25]. The purpose of our study is to emphasize the role of DWI in the preoperative staging of cervical carcinoma.

In this study, the age at presentation for cervical carcinoma was ranged between 33 and 82 years (mean 56 years). Postmenopausal vaginal bleeding was seen in 38 patients (52.6%), representing the most common presenting symptom. It was followed by irregular and heavy vaginal bleeding in perimenopausal women (eight patients, 14%) and irregular vaginal bleeding in women in the reproductive age group (19 patients, 33.4%). The most common histopathology of cervical cancer in this study was SCC (in situ carcinoma in 5.3%, invasive keratinizing in 59.6%, and invasive non-keratinizing in 22.8% of the cases), followed by endocervical adenocarcinoma (12.3% patients). The FIGO stages of the patients included were IB in 21% of patients, IIA in 5.3%, IIB in 12.3%, IIIA in 1.8%, IIIC in 21.1%, IVA in 22.8%, and IVB in 15.8%.

A study by Mansour, et al. included patients aged between 30 and 80 years (mean 49 years) diagnosed with cervical carcinoma and also stated that the most common histopathology was SCC, demonstrated in 72% of the cases [26]. Other types of tumors consisted of adenocarcinoma (12%), sarcomatoid cervical carcinoma (4%), spindle cell tumor (4%), basaloid carcinoma (4%) and undifferentiated carcinoma (4%). The FIGO stages of these cases were: stage IB (n=22, 44%), stage IIA (n=8, 16%), stage IIB (16%), stage IIIB (n=10, 20%), stage IVA (n=1, 2%), and stage IVB (n=1, 2%).

We performed an assessment of the aspect of the tumor on T2 weighed images and it appeared hypointense in 29.8%, isointense in 31.6%, hyperintense in 10.5%, and heterogenous in 28.1% of the cases. Cervical cancer presents a high signal on contrast-enhanced MRI compared to the lower signal of the cervical tissue. Usually, cervical tumors are avidly enhanced in the early phases, permitting the distinction between recurrent lesions from radiation fibrosis [27]. In the present study, cervical cancer showed heterogeneous enhancement in 49.1%, while low contrast enhancement was found in 50.9% of cases. A study by Abd Elsalam et al. has found heterogenous enhancement in 57% of the cases [28].

In the present study, malignant cervical tissue showed restricted diffusion and low ADC values relative to normal tissue. The mean ADC value for malignant lesions was 0.79 × 10−3 mm^2^/second, while the mean ADC value for normal cervical tissue was 1.59 × 10−3 mm^2^/second. The ADC mean showed lower values with increased FIGO stage of the tumor. A study by Liu et al. also demonstrated a decreasing trend in the ADC mean value with higher FIGO stages [29]. The ADC mean value in late-stage disease (IIIC1) was significantly lower than in locally advanced (IB3, IIA2) stages (P<0.001), which was again significantly lower than in early stages (IB1, IB2, IIA1) (P<0.001). The current study also showed statistically significant variation in ADC mean value according to histopathological type, differentiation grade, extension to surrounding tissues, size, and pelvic lymph node metastasis.

Abd Elsalam et al. showed that the mean ADC value of malignant tissue was 0.82 × 10−3 ± 0.1 SD mm^2^/second and that the mean ADC value in the control group was 1.56 × 10-3 mm^2^/second) [28]. They also reported no statistically significant difference in the ADC mean value of different histopathological types. The ADC mean value was 0.84 × 10−3 mm^2^/second for keratinizing SCC, 0.76 × 10−3 mm^2^/second for non-keratinizing SCC, and 0.87 × 10−3 mm^2^/second for papillary adenocarcinoma. Kuang et al. showed that ADC mean value in malignant cervical tumors measured 0.916 × 10−3 ± 0.15 SD mm^2^/second, while benign lesions had higher values, 1.396 × 10−3 ± 0.15 SD mm^2^/second in cervical leiomyomas and 1.426 × 10−3 ± 0.11 SD mm^2^/second in cervical polyps [30]. Nakamura et al. reported that the mean ADC value in the patients with cervical cancer they evaluated was 0.852 × 10−3 mm^2^/second. They also stated that well-differentiated tumors had higher ADC mean values than poorly differentiated tumors (1.2 × 10−3 mm^2^/second vs. 1.1 × 10−3 mm^2^/second) (P = 0.01) [31]. 

There are some limitations that should be considered as they may influence the ADC mean value of the lesions. The tumors may overlap with benign conditions such as inflammation and fibrosis that may also show decreased ADC values, potentially leading to overstaging the tumor. ADC values can be influenced by the MRI equipment, imaging protocols and post-processing techniques. Standardization is necessary for reliable comparison across studies and institutions. In order to integrate ADC mean value assessment into routine clinical practice for cervical cancer, multidisciplinary collaboration is essential. Radiologists, oncologists, and radiotherapists need to work together to integrate de ADC values into patient management strategies. Standardization of imaging protocols and continuous education on the interpretation of ADC maps are also critical. Overall, the ADC mean value provides a non-invasive, quantitative tool that enhances the management of cervical cancer, from diagnosis to post-treatment follow-up.

## Conclusions

Our study confirms the high accuracy of MRI, especially using DWI and ADC mapping in the preoperative staging of cervical cancer. This study demonstrates that DWI improves the accuracy of diagnosing and staging cervical cancer compared to traditional imaging techniques, including a better differentiation between benign and malignant lesions, a more precise tumor characterization, and an improved detection of small or early-stage tumors. 

In the future, diagnosing and staging cervical cancer will improve comprehensive MRI protocols that include DWI in both axial and sagittal acquisition and may offer more detailed information about the extension of the lesions. Our findings suggest that including the mean ADC value in the primary evaluation of the tumor could serve as an indicator of the aggressiveness and risk of recurrence of cervical cancer.
